# The Frequency of Infective Endocarditis in *Candida* Bloodstream Infections: a Retrospective Study in a Child Hospital

**DOI:** 10.21470/1678-9741-2017-0049

**Published:** 2018

**Authors:** Ahu Kara, İlker Devrim, Timur Meşe, Nuri Bayram, Murat Yılmazer, Gamze Gülfidan

**Affiliations:** 1 Department of Pediatric Infectious Diseases, Dr. Behçet Uz Children's Hospital, İzmir, Turkey.

**Keywords:** Infection, Candida, Endocarditis

## Abstract

**Introduction:**

Fungal endocarditis is reported less frequently than bacterial endocarditis, with an incidence of 0-12% of the total pediatric infective endocarditis.

**Objective:**

In this study, the incidence of infective endocarditis in *Candida* bloodstream infections in a tertiary hospital during the periods of 2007 and 2016 was reviewed.

**Methods:**

Patients with positive blood or catheter cultures in terms of *Candida spp.* during the study period of January 2007 and January 2016 were analyzed in terms of *Candida* infective endocarditis. Infective endocarditis was defined according to the modified Duke criteria. The outcome, possible associated predisposing factors for *Candida* endocarditis were determined.

**Results:**

221 patients and 256 attacks with positive blood or catheter cultures in terms of *Candida* were included in the study. The most common *Candida* species was *Candida parapsilosis*, isolated in 157 (61.3%) attacks, followed by *Candida albicans* in 70 (27.3%). Neurological diseases (23%), hemato-oncological diseases (12.1%), previously known heart diseases (8.2%), inborn errors of metabolism (9%) were common comorbidities. Twelve (5.4%) patients had a previous history of cardiac surgery. Among the 221 patients, *Candida endocarditis* was present in only two (0.9%) of them.

**Conclusion:**

Although *Candida* infective endocarditis is an uncommon but frequently fatal infection in pediatrics, echocardiography should be performed routinely for patients with positive blood or catheter cultures in terms of *Candida*. Prompt and effective antimicrobial therapy might prevent cardiac surgery in selected cases, however this could not be a general rule for all patients.

**Table:** 

Abbreviations, acronyms & symbols				
ASD	= Atrial septal defect		MR	= Mitral regurgitation
BSIs	= Bloodstream infections		PE	= Pericardial effusion
CMP	= Cardiomyopathy		PFO	= Patent foramen ovale
CVC	= Central venous catheter		PS	= Pulmonary stenosis
ESCMID	= European Society of Clinical Microbiology and Infectious Diseases		SPSS	= Statistical Package for the Social Sciences
FE	= Fungal endocarditis		TEE	= Transesophageal echocardiography
ICUs	= Intensive care units		TGA	= Transposition of the great arteries
IDSA	= Infectious Diseases Society of America		TOF	= Tetralogy of Fallot
IE	= Infective endocarditis		TR	= Tricuspid regurgitation
			VSD	= Ventricular septal defect

## INTRODUCTION

Infective endocarditis (IE) is the most common and fatal form of endovascular infections. Fungal endocarditis (FE) is reported less frequently than bacterial endocarditis, with an incidence of 0-12% of the total pediatric IE admissions^[1,2]^. *Candida* species were reponsible of the two thirds of FE. *Candida* IE is a rare and poorly understood complication of fungemia. Prolonged fever and changing heart murmur are the most common clinical manifestations. The most frequently reported risk factors for FE are previous surgery, indwelling foreign bodies such as catheters, antibiotic use, underlying heart disease, prosthetic valves, and immunocompromising conditions^[1,3,4]^. The recommended treatment of *Candida* IE is an amphotericin B-based regimen plus surgical intervention, often followed by long-term fluconazole for suppression^[5]^. Despite aggressive antifungal and surgical therapy, mortality from *Candida* endocarditis was reported between 30-80%^[6-12]^. Therefore, clinicians should be alert for early diagnosis and prompt treatment of FE.

The aim of this study was to determine the incidence of *IE* in *Candida* bloodstream infections (BSIs) in a tertiary hospital during the periods of 2007 and 2016.

## METHODS

### Study Design

Data for this study were derived from hospitalized patients between January 2007 and January 2016 in Dr Behçet Uz Children Diseases and Surgery Training and Research Hospital. Demographic data included age, gender, patients ward, echocardiography findings, presence of an indwelling central venous catheter (CVC), cause of hospitalization, presence of underlying disease, type of *Candida* in positive blood and/or catheter cultures, presence of fluconazole prophylaxis, presence of previous cardiac surgery and prosthetic valve, presence of mechanical ventilation. Data were recorded from medical records.

### Study Population

Patients were included in this study if they had two or more positive blood or catheter cultures in terms of *Candida* If the same patient had an attack of candidemia again at least 3 weeks after 3 consecutive negative blood or catheter cultures for *Candida*, this was accepted as a new attack.

### Definitions

*IE* was defined according to the modified Duke criteria^[13,14]^.

A definitive clinical diagnosis was done based on the following criteria: 2 major criteria or 1 major criteria and 3 minor criteria or 5 minor criteria (Table 1).

**Table 1:** Modified Duke criteria.

Major blood culture criteria	Major echocardiographic criteria	Minor criteria
• Two blood cultures positive for organisms typically found in patients with IE • Blood cultures persistently positive for one of these organisms, from cultures drawn more than 12 hours apart • Three or more separate blood cultures drawn at least 1 hour apart	• Echocardiogram positive for IE, documented by an oscillating intracardiac mass on a valve or on supporting structures, in the path of regurgitant jets, or on implanted material, in the absence of an alternative anatomic explanation • Myocardial abscess • Development of partial dehiscence of a prosthetic valve • New-onset valvular regurgitation	• Predisposing heart condition or intravenous drug use • Fever of 38ºC (100.4ºF) or higher • Vascular phenomenon, including major arterial emboli, septic pulmonary infarcts, mycotic aneurysm, intracranial hemorrhage, conjunctival hemorrhage, or Janeway lesions • Immunologic phenomenon such as glomerulonephritis, Osler nodes, Roth spots, and rheumatoid factor • Positive blood culture results not meeting major criteria or serologic evidence of active infection with an organism consistent with IE • Echocardiogram results consistent with IE but not meeting major echocardiographic criteria

### Statistical Analysis

All statistical analyses were performed using Statistical Package for the Social Sciences version 18.0 (SPSS, Microsoft Inc., Chicago, Il, USA). Patients' demographics and clinical variables were described as mean, median (if not normally distributed), and standard deviation for continuous data and proportions for nominal and ordinal data.

## RESULTS

Two hundred and twenty-one patients and 256 attacks with positive blood or catheter cultures in terms of *Candida* were included in the study. One hundred and twenty-nine (58.4%) of patients were male, 92 (41.6%) were female. The median age of the patients was 10.0 months (ranging from 7 days of age to 17 years). One hundred and eighty-six (72.6%) attacks had been observed in patients hospitalized in intensive care units (ICUs) and 70 (27.4%) attacks in our study in other wards in stead of ICUs.

**Fig. 1:**
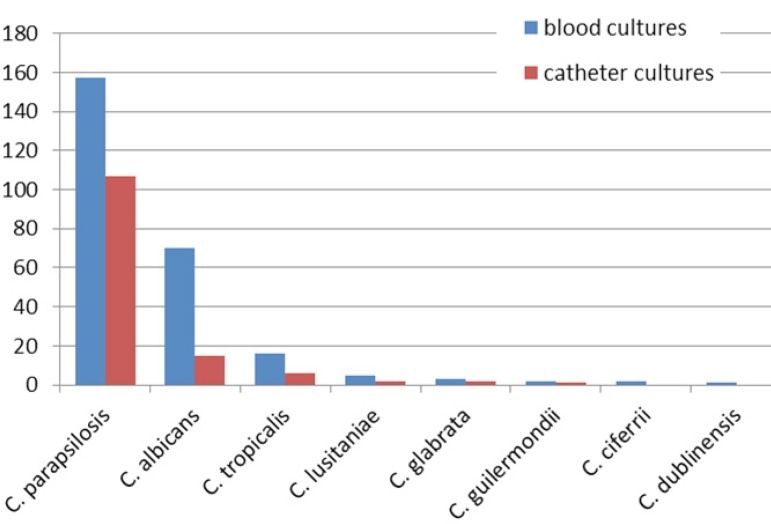
Distribution according to isolated Candida species in blood cultures and catheter cultures.

Most common *Candida* species was *Candida parapsilosis* isolated in 157 (61.3%) attacks followed by *Candida albicans* in 70 (27.3%), *Candida tropicalis* in 16 (6.3%), *Candida lusitaniae* in five (2%), *Candida glabrata* in three (1.2%), *Candida guilermondii* in two (0.8%), *Candida ciferrii* in two (0.8%) and *Candida dublinensis* in one (0.4%). Distribution according to types of *Candida* in blood cultures was reviewed in Figure 1. The catheter-related BSIs were present in 133 attacks. One hundred and seven (80.4%) patients were positive for *Candida parapsilosis*, 15 (11.2%) for *Candida albicans*, six (4.5%) for *Candida tropicalis*, two (1.5%) for *Candida lusitaniae*, two (1.5%) for *Candida glabrata* and one (0.7%) for *Candida guilermondii*. Distribution according to types of *Candida* in catheter cultures was reviewed in Figure 1.

An indwelling CVC was present in 117 (47.1%) patients. Seventy-six (29.7%) patients were receiving fluconazole prophylaxis at the time of positive blood or catheter culture during attacks. Among 221 patients, 12 (5.4%) had cardiac surgery, including complex intracardiac repairs (7), palliative shunt procedures (4) and prosthetic valve (1). Approximately half of the patients (52.3%) had underlying diseases, including 59 (23%) neurological diseases, 31 (12.1%) hemato-oncological diseases, 21 (8.2%) previously known congenital heart diseases, and 23 (9%) inborn errors of metabolism.

**Fig. 2:**
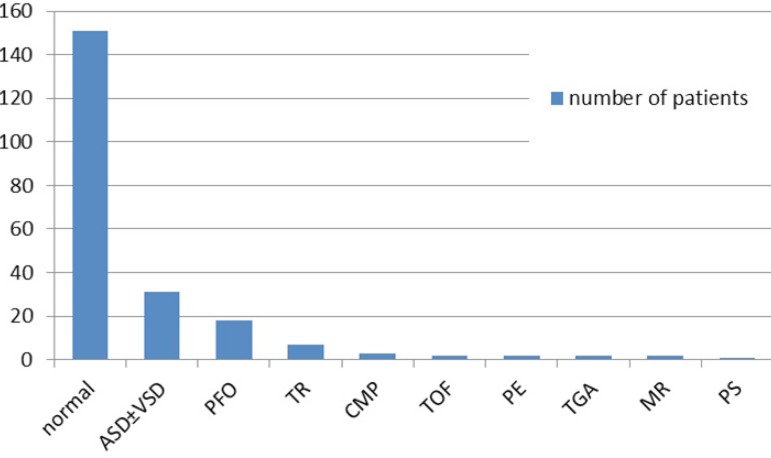
Distribution according to echocardiography. ASD±VSD=atrial septal defect and/or ventricular septal defect; CMP=cardiomyopathy; MR=mitral regurgitation; normal: normal echocardiogram findings; PE=pericardial effusion; PFO=patent foramen ovale; PS=pulmonary stenosis; TGA=transposition of the great arteries; TOF=tetralogy of Fallot; TR=tricuspid regurgitation

Echocardiography had been performed in all patients. In echocardiography, normal cardiac findings were present in 151 (68.4%) patients, while 31 (14.8%) patients had atrial septal defect (ASD) and/or ventricular septal defect (VSD), 18 (8.1%) patients had patent foramen ovale (PFO) and seven (3.2%) had tricuspid regurgitation (TR). More rare findings were cardiomyopathy (CMP) in three (1.4%), tetralogy of Fallot (TOF) in two (0.9%), pericardial effusion (PE) in two (0.9%), transposition of the great arteries (TGA) in two (0.9%), mitral regurgitation (MR) in two (0.9%), and pulmonary stenosis (PS) in one (0.5%) patient. The distribution according to the echocardiography was summarized in Figure 2.

Among the 221 patients, *Candida* endocarditis was detected in only two (0.9%) patients. One of the patients was a 9-yearold boy with cerebral palsy who was presented at our hospital with cough, tachypnea and fever. He was admitted to the ICU with severe pneumonia. On the 14^th^ day of his treatment, echocardiography revealed an echogenic mass of 50x40 mm attached to the wall of the left internal jugular vein. *Candida albicans* was isolated from two consecutive blood cultures. Meropenem, vancomycin and amphotericin B therapy were initiated for the treatment of IE. *C. albicans* was reported as susceptible to caspofungin, amphotericin B and fluconazole. Cardiac surgery was planned due to the patient's status. However, due to the response to antifungal medical treatment and improvement in his clinic and the recovery of intracardiac mass, surgical treatment was postponed.

The other patient was a 5-month-old girl who was presented to our hospital with dyspnea and high-grade fever. She had a medical history of secundum ASD and perimembranous VSD which had been closed with the Amplatzer(tm) Septal Occluder with angiography 2 months ago. She was transferred into pediatric ICU and her first echocardiography revealed pulmonary hypertension and the subclavian venous catheter was inserted into the patient. Her first blood culture was sterile. On the fifth day of treatment, two-dimensional echocardiography showed a 30x25 mm heterogenous mass attached to the right atrium wall. Piperacillintazobactam, vancomycin and gentamicin therapy were initiated for the treatment of IE. *Candida parapsilosis* was isolated from two different blood and CVC cultures. *C. parapsilosis* has been reported to be susceptible to caspofungin, amphotericin B and fluconazole, and caspofungin was initiated as the initial antifungal therapy. On the seventh day of the antifungal therapy, the fever returned and the consecutive echocardiography revealed a slowly resolving mass. Due to the improved patient status, cardiac surgery was delayed. On the 45^th^ day of antifungal therapy, the echocardiography revealed complete resolution and the patient was discharged from the hospital. After discharge, the patient received a long-term (6 weeks) intermittent suppressive oral fluconazole (9 mg/kg/day) therapy.

## DISCUSSION

Fungemia rates have increased significantly in recent years, resulting in a growing number of populations at risk for this disease^[9,15]^. Fungal endocarditis is an uncommon infection and predominantly caused by *Candida* species, and less frequently by *Aspergillus* species. Invasive candidiasis was a major cause of morbidity and mortality in children. Historically, *C. albicans* has been the most common isolate from fungal BSIs^[16]^. Recently, there has been a reported increase in the incidence of nonalbicans *Candida spp.*, especially of *C. parapsilosis* ^[17-21]^, whose incidence could change according to hospitals. According to our data in the study, C*. parapsilosis* was the most dominant candida in *Candida* BSIs.

FE has been reported to cause 0-12% (average 1.1%) of the total IE cases in children worldwide. The incidence rate is approximately 1.5-4 cases per 10 million children^[22]^. In our hospital, 15 patients with IE had been identifed according to modified Duke criteria during the study period. As a result, the ratio of FE cases in all IE cases diagnosed in our hospital was 13.3%, which is slightly higher than the literature. Puig-Asensio et al.^[23]^ reported that incidence of IE in *Candida* BSIs was 1.9% (14/512 patients with candidemia), which was relatively high compared to our findings. The reported cases of FE were less than a few hundred in patients of any age, while two thirds of FE were associated with *Candida* species. *Candida* IE is an uncommon but frequently fatal infection in pediatrics with the survival rate remains below 25%^[1,10]^.

Our patients have multiple possible risk factors for developing FE, including prolonged ICU stay, prolonged use of intravenous antibiotics, underlying heart disease (ASD and VSD), previous cardiac surgery, and presence of CVC. In addition, immunocompromising conditions were well-defined risk factors for developing FE^[1,10,11]^. Multifactorial risk factors in a single patient might be more likely to cause FE^[24]^. Repaired congenital heart disease with residual defects and the first six months of repaired congenital heart disease with no residual defects are at risk of IE^[9]^. The second patient's heart defects were closed with Septal Occluder with angiography 2 months ago. Ağın et al.^[3]^ reported in their study the longer pediatric ICU stays, mechanical ventilation, CVC, total parenteral nutrition were the risk factors for the development of *Candida* infections in pediatric ICU. However, studies for risk factors especially for FE were limited, while risk factors for *Candida* BSIs were well-defined.

FE typically occurs in otherwise critically ill patients and is often part of a confusing clinical picture, with most patients having difficulty meeting the Duke criteria for IE^[25]^. Transthoracic echocardiography is less sensitive than transesophageal echocardiography (TEE), but is also less invasive. Intracardiac vegetations and thrombi are the most common types, but are still rare^[9,22]^. Although these findings have rarely been observed, both of our patients had echogenic masses which could be visualized with transthoracic echocardiography.

The guidelines of Infectious Diseases Society of America (IDSA) and European Society of Clinical Microbiology and Infectious Diseases (ESCMID) for treatment of *Candida* IE recommend either an amphotericin B-based regimen or an echinocandin-based regimen, both in combination with adjunctive surgical therapy if possible^[26-28]^. Fluconazole therapy has been less successful than other agents^[27]^. In almost all reported cases of survival, surgical management was necessary to supplement antifungal medical therapy. Surgery is mandatory in the majority of cases and it is agreed that it should be performed as early as possible^[26]^. In one patient with IE, the surgery was planned, however, the surgical treatment was delayed due to recovery with medical antifungal treatment. Current guidelines for endocarditis recommend initial or induction therapy with Amphotericin B with or without flucytosine combined with surgical removal of vegetation, followed by chronic suppressive therapy with oral fluconazole^[29,30]^.

*Candida* IE is associated with a high mortality rate that was not affected by the choice of antifungal therapy or by adjunctive surgical intervention^[31]^. Recently published article by Arnold et al.^[32]^ demonstrated that mortality did not differ between those undergoing surgical therapy and those receiving medical therapy alone. In another study, among 33 cases derived from the International Collaboration of Endocarditis-Prospective Cohort Study, the mortality rate was similar whether surgery was performed or not^[8]^. In contrast, Lefort et al.^[9]^ suggested that early cardiac surgery during *Candida* IE should always be attempted, and only patients with very poor medical status might not be operated on according to the findings in their study.

## CONCLUSION

FE is often difficult to diagnose, therefore, echocardiography should be performed routinely for patients with positive blood or catheter cultures in terms of *Candida* and TEE should be performed in the presence of underlying clinical risk factors and high clinical suspicions.

**Table:** 

Authors' roles & responsibilities	
AK	Concept, acquisition, analysis, interpretation of data for the work; final approval of the version to be published
İD	Concept, acquisition, analysis, interpretation of data for the work; revising; final approval of the version to be published
TM	Analysis; final approval of the version to be published
NB	Analysis; final approval of the version to be published
MY	Revising; final approval of the version to be published
GG	Final approval of the version to be published

## References

[1] Ellis ME, Al-Abdely H, Sandridge A, Greer W, Ventura W (2001). Fungal endocarditis: evidence in the world literature,
1965-1995. Clin Infect Dis.

[2] Millar BC, Jugo J, Moore JE (2005). Fungal endocarditis in neonates and children. Pediatr Cardiol.

[3] Ağın H, Devrim I, İşgüder R, Karaarslan U, Kanık E, Günay I (2014). Risk factors for candidemia in pediatric intensive care unit
patients. Indian J Pediatr.

[4] Horn DL, Neofytos D, Anaissie EJ, Fishman JA, Steinbach WJ, Olyaei AJ (2009). Epidemiology and outcomes of candidemia in 2019 patients: data
from the prospective antifungal therapy alliance registry. Clin Infect Dis.

[5] Pappas PG, Rex JH, Sobel JD, Filler SG, Dismukes WE, Walsh TJ (2004). Guidelines for treatment of candidiasis. Clin Infect Dis.

[6] Nazarian M, McCullough GH, Fielder DL (1976). Bacterial endocarditis in pregnancy: successful surgical
correction. J Thorac Cardiovasc Surg.

[7] Baddley JW, Pappas PG (2005). Antifungal combination therapy: clinical
potential. Drugs.

[8] Baddley JW, Benjamin Jr DK, Patel M, Miró J, Athan E, Barsic B, International Collaboration on Endocarditis-Prospective Cohort Study
Group (ICE-PCS) (2008). Candida infective endocarditis. Eur J Clin Microbiol Infect Dis.

[9] Lefort A, Chartier L, Sendid B, Wolff M, Mainardi JL, Podglajen I, French Mycosis Study Group (2012). Diagnosis, management and outcome of Candida
endocarditis. Clin Microbiol Infect.

[10] Pierrotti LC, Baddour LM (2002). Fungal endocarditis, 1995-2000. Chest.

[11] Benjamin Jr DK, Miro JM, Hoen B, Steinbach WJ, Fowler Jr VG, Olaison L, ICE-MD Study Group (2004). Candida endocarditis: contemporary cases from the International
Collaboration of Infectious Endocarditis Merged Database
(ICE-mD). Scand J Infect Dis.

[12] Falcone M, Barzaghi N, Carosi G, Grossi P, Minoli L, Ravasio V, Italian Study on Endocarditis (2009). Candida infective endocarditis: report of 15 cases from a
prospective multicenter study. Medicine (Baltimore).

[13] Li JS, Sexton DJ, Mick N, Nettles R, Fowler Jr VG (2000). Proposed modifications to the Duke criteria for the diagnosis of
infective endocarditis. Clin Infect Dis.

[14] Durack DT, Lukes AS, Bright DK (1994). New criteria for diagnosis of infective endocarditis: utilization
of specific echocardiographic findings. Duke Endocarditis
Service. Am J Med.

[15] Martin GS, Mannino DM, Eaton S, Moss M (2003). The epidemiology of sepsis in the United States from 1979 through
2000. N Engl J Med.

[16] Chang A, Neofytos D, Horn D (2008). Candidemia in the 21st century. Future Microbiol.

[17] Roilides E, Farmaki E, Evdoridou J, Dotis J, Hatziioannidis E, Tsivitanidou M (2004). Neonatal candidiasis: analysis of epidemiology, drug
susceptibility, and molecular typing of causative isolates. Eur J Clin Microbiol Infect Dis.

[18] Celebi S, Hacimustafaoglu M, Ozdemir O, Ozkaya G (2008). Nosocomial candidaemia in children: results of a 9-year
study. Mycoses.

[19] Pfaller MA, Diekema DJ (2007). Epidemiology of invasive candidiasis: a persistent public health
problem. Clin Microbiol Rev.

[20] Conde-Rosa A, Amador R, Pérez-Torres D, Colón E, Sánchez-Rivera C, Nieves-Plaza M (2010). Candidemia distribution, associated risk factors, and attributed
mortality at a university-based medical center. P R Health Sci J.

[21] Zaoutis T (2010). Candidemia in children. Curr Med Res Opin.

[22] Baltimore RS, Jenson HB, Baltimore RS (2002). Infective endocarditis. Pediatric ınfectious diseases: principles and practice.

[23] Puig-Asensio M, Padilla B, Garnacho-Montero J, Zaragoza O, Aguado JM, Zaragoza R (2014). Epidemiology and predictive factors for early and late mortality
in Candida bloodstream infections: a population-based surveillance in
Spain. Clin Microbiol Infect.

[24] Simon MS, Somersan S, Singh HK, Hartman B, Wickes BL, Jenkins SG (2014). Endocarditis caused by Rhodotorula infection. J Clin Microbiol.

[25] Alhaji M, Sadikot RT (201). Cryptococcal endocarditis. South Med J.

[26] Cornely OA, Bassetti M, Calandra T, Garbino J, Kullberg BJ, Lortholary O, ESCMID Fungal Infection Study Group (2012). ESCMID guideline for the diagnosis and management of Candida
diseases 2012: non-neutropenic adult patients. Clin Microbiol Infect.

[27] Smego Jr RA, Ahmad H (2011). The role of fluconazole in the treatment of Candida endocarditis:
a meta-analysis. Medicine (Baltimore).

[28] Pappas PG, Kauffman CA, Andes D, Benjamin Jr DK, Calandra TF, Edwards Jr JE, Infectious Diseases Society of America (2009). Clinical practice guidelines for the management of candidiasis:
2009 update by the Infectious Diseases Society of America. Clin Infect Dis.

[29] Devathi S, Curry B, Doshi S (2014). Isolated pulmonary valve infective endocarditis in a middle aged
man caused by Candida albicans: a case report. BMC Infect Dis.

[30] Yuan SM (2016). Fungal endocarditis. Braz J Cardiovasc Surg.

[31] Steinbach WJ, Perfect JR, Cabell CH, Fowler VG, Corey GR, Li JS (2005). A meta-analysis of medical versus surgical therapy for Candida
endocarditis. J Infect.

[32] Arnold CJ, Johnson M, Bayer AS, Bradley S, Giannitsioti E, Miró JM (2015). Candida infective endocarditis: an observational cohort study
with a focus on therapy. Antimicrob Agents Chemother.

